# Sulfur‑Containing Additives for Enhanced Kinetics and Interfacial Stability of Phosphorus Anodes in Li‑Ion Batteries

**DOI:** 10.1002/advs.75457

**Published:** 2026-05-25

**Authors:** Huixian Xie, Xinze Li, Zhenjiang Yu, Hongyi Chen, Lingwen Liu, Kwun Nam Hui

**Affiliations:** ^1^ Joint Key Laboratory of the Ministry of Education Institute of Applied Physics and Materials Engineering University of Macau Macau SAR P. R. China; ^2^ Department of Chemistry Lancaster University Lancaster UK

**Keywords:** fast‐charging kinetics, interfacial dynamics, lithium‐ion battery, phosphorus‐based anode, SEI formation

## Abstract

Red phosphorus offers ultrahigh theoretical capacity for lithium‐ion battery anodes but is fundamentally limited by poor electronic conductivity, sluggish lithium‐ion diffusion, and severe volume expansion that destabilizes the solid‐electrolyte interphase. Here, we introduce a synergistic strategy that simultaneously regulates reaction kinetics and interfacial stability using sulfur‐containing additives, lithium sulfide, and in situ generated lithium sulfate. Lithium sulfide confines lithium polyphosphide intermediates, enabling a direct and highly reversible conversion reaction, while lithium sulfate selectively absorbs solvent molecules and directs the formation of a uniform, lithium‐oxide‐dominated and mechanically adaptive interphase. Through this cooperative modulation, reaction kinetics are accelerated, structural integrity is preserved during cycling, and interfacial failure is effectively suppressed. As a result, the optimized red phosphorus anode retains 96.1% and 94.1% of its capacity after 450 and 1000 cycles at 1 and 4 C respectively, and still maintains 80% capacity retention after 1200 cycles at 6 C. This dual‐functional strategy establishes a coherent design principle for durable, high‐capacity conversion‐type anodes.

## Graphical abstract

A novel synergistic strategy by employing dual functional sulfur‐containing additives of lithium sulfide (Li_2_S) and its oxidation product lithium sulfate (Li_2_SO_4_) was proposed. Li_2_SO_4_ absorbs the solvent molecules to direct the formation of a uniform, Li_2_O‐rich, and adaptive interphase, which accommodates volume expansion and suppresses parasitic reactions. Li_2_S confines lithium polyphosphides (LiPPs) during reaction to promote the reaction kinetics.

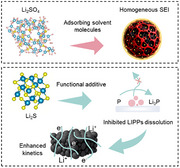

## 1 Introduction

Red phosphorus (RP) is a highly attractive anode material for lithium‐ion batteries (LIBs) owing to its ultrahigh theoretical capacity (2596 mAh g^−^^1^), earth abundance, and low cost [1]. However, its practical implementation remains severely constrained by persistent kinetic and interfacial limitations. RP exhibits extremely low intrinsic electronic conductivity (∼10^−10^ S cm^−1^), sluggish lithium‐ion diffusion, and slow reaction kinetics associated with the dissolution of lithium polyphosphides (LiPPs) [2]. In addition, RP undergoes severe volume expansion (>300%) during lithiation, leading to pronounced interfacial failure [3, 4]. These intrinsically coupled issues result in large polarization, repeated fracture of the solid‐electrolyte interphase (SEI), continuous electrolyte decomposition, and progressive structural degradation during cycling [5].

Extensive strategies including carbon coating [6], nanostructuring [7], polymeric binders [8], prelithiation [9], amorphization [10], adsorbent [11], and electrolyte engineering [12], have been explored to mitigate individual challenges of RP anodes, such as poor conductivity, large volume change, LiPPs dissolution, low initial Coulombic efficiency, and unstable SEI formation. Despite these advances, a durable and high‐performance RP anode has not yet been realized in a synergistic manner. The fundamental limitation arises from the fact that most existing approaches address these issues in isolation, lacking a coordinated mechanism to resolve the intrinsic coupling between charge transport, reaction kinetics, and interfacial stability. For example, conductive carbon networks improve electron transport but dilute volumetric energy density and do not fundamentally accelerate lithium‐ion kinetics [13]. Porous nanostructures can buffer volume expansion but significantly increase surface area, thereby exacerbating parasitic interfacial reactions [14]. Electrolyte regulation may promote SEI formation, yet often produces compositionally heterogeneous and mechanically fragile interphases that readily crack under repeated volume fluctuations [15, 16]. As a result, these strategies fall short of providing a holistic solution that simultaneously optimizes reaction kinetics and interfacial robustness.

**FIGURE 1:**
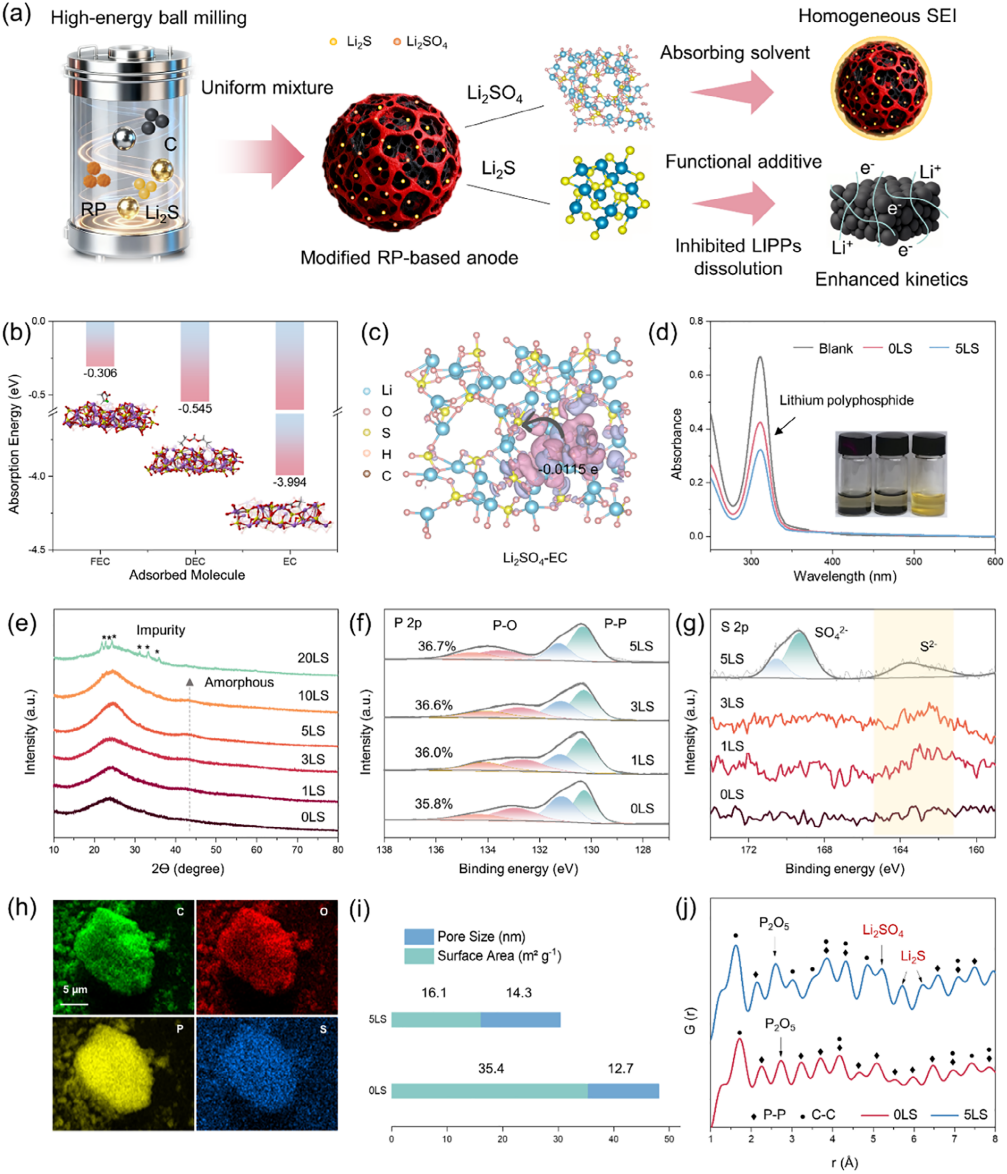
(a) Schematic illustration of the reaction and interface engineering mechanism in the modified RP anode. (b) Absorption energies of FEC, DEC, and EC on the Li_2_SO_4_ surface. The inset is the optimized adsorption configurations. (c) Charge density difference maps showing the interfacial electron redistribution between EC and Li_2_SO_4_ surface (Purple: electron accumulation, pink: electron depletion). (d) UV–vis absorption spectra and corresponding optical images. (e) XRD patterns of RP composites with varying Li_2_S contents. (f) P 2p and (g) S 2p XPS spectra of samples containing different amounts of Li_2_S. (h) EDS elemental mapping of the 5LS sample showing uniform elemental distribution. (i) Pore size and surface area of 0LS and 5LS. (j) PDF curves of the 0 and 5LS samples. The diamond symbol represents P─P correlations and the dot symbol denotes C─C correlations.

To address this long‐standing challenge, we propose a synergistic dual‐functional strategy based on sulfur‐containing additives, lithium sulfide (Li_2_S) and its oxidation derivative lithium sulfate (Li_2_SO_4_), to concurrently regulate reaction kinetics and interfacial stability in RP anodes. The key innovation lies in the complementary roles of these two components (Figure 1a). Li_2_S acts as a LiPPs confinement additive, suppressing the dissolution and diffusion of LiPPs and retaining them at the reaction interface, thereby enabling a more complete and reversible conversion reaction toward the final Li_3_P phase. Simultaneously, Li_2_SO_4_ functions as an interphase regulator, directing the formation of a uniform, lithium‐oxide‐rich (Li_2_O‐rich) and mechanically adaptive SEI that accommodates large volume changes and suppresses parasitic reactions. Through this coupled modulation, reaction kinetics and interfacial stability are simultaneously enhanced. The optimized RP anode delivers a high initial Coulombic efficiency (ICE) of 88.6% and a reversible capacity of 920.5 mAh g^−1^ at 1 C, while maintaining 96.1% capacity retention after 450 cycles. Exceptional rate capability and durability are further demonstrated by 94.1% capacity retention after 1000 cycles at 4 C and 80% retention after 1200 cycles at 6 C. Even when assembled into a full‐cell configuration, the electrode retains 76.4% of its capacity after 500 cycles. These results establish a unified design principle for simultaneously overcoming kinetic and interfacial limitations in conversion‐type RP anodes, highlighting their strong potential for next‐generation high‐energy lithium‐ion batteries.

## 2 Results and Discussion

### 2.1 Material Properties and Characterization

In this work, sulfur‐containing additives were introduced into an RP anode through a high‐energy ball‐milling process, as illustrated in Figure 1a. The resulting composites are designated as 0, 1, 3, 5, 10, and 20LS, corresponding to Li_2_S additions of 0, 1, 3, 5, 10, and 20 wt.%, respectively. During high‐energy milling, Li_2_S partially oxidizes and decomposes to form Li_2_SO_4_, which becomes uniformly dispersed within both the bulk and surface regions of the composite. To clarify the oxidizing source, raw materials, including RP and conductive carbon, the manually mixed (non‐ball‐milled) 0LS‐M sample and a hand‐ground sample containing 5 wt.% Li_2_S (denoted 5LS‐M) was analyzed for comparison. X‐ray photoelectron spectroscopy (XPS) analysis of the raw materials shows that both pristine RP and conductive carbon contain surface oxygen species, including P─O and C─O/C═O groups (Figures S1 and S2). These results indicate that the starting materials are not oxygen‐free and may serve as the main oxygen source during the subsequent synthesis process. Control samples prepared without ball milling show similar oxygen‐related features, while ball milling induces pronounced changes only in the presence of Li_2_S (Figure S3), suggesting that pre‐existing oxygen on the raw materials, rather than trace air exposure or jar‐derived impurities, is the main contributor to the observed oxidation.

The role of the in situ generated Li_2_SO_4_ in guiding interphase formation was first investigated. As shown in Figure 1b, the absorption energies of Li_2_SO_4_ for three carbonate molecules (fluoroethylene carbonate, FEC; diethyl carbonate, DEC; ethylene carbonate, EC) were calculated. The strongest absorption of EC on Li_2_SO_4_ surface implies that EC tends to be preferentially decomposed at the surface contributing to an SEI rich in lithium carbonate (Li_2_CO_3_) and Li_2_O [17, 18]. This is further supported by the significant electron transfer (−0.0115 e) and pronounced charge redistribution observed at the Li_2_SO_4_‐EC interface (Figure 1c; Figure S4), suggesting EC's dominant role in forming a stable chemisorbed layer. With the Li_2_SO_4_ being uniformly distributed in the 5LS sample, this allows for the formation of a homogeneous SEI layer, which is an essential feature for long‐term interfacial stability. Concurrently, the function of Li_2_S in inhibiting the dissolution of LiPPs was examined. Consistent with the prior report [19], Li_2_S demonstrates a higher absorption energy for LiPPs than for solvent molecules, confirming its ability to suppress LiPPs dissolution. Static adsorption experiments (Figure 1d) directly assessed this affinity, revealing that the 5LS sample possesses the greatest capacity for LiPPs absorption. This effective confinement of LiPPs at the reaction promotes a more complete and reversible conversion of RP to Li_3_P. To probe the structural and chemical consequences of this additive strategy, detailed characterizations were performed. X‐ray diffraction (XRD) patterns show that all samples except 20LS are predominantly amorphous (Figure 1e), whereas 20LS exhibits additional crystalline peaks attributed to excess Li_2_S. XPS was further conducted on 0, 1, 3, and 5LS to investigate surface chemical evolution. In the P 2p spectra, peaks at 130.3 and 131.1 eV correspond to P─P bonds (P 2p_3/2_ and P 2p_1/2_) [20], while doublets at 133.0 and 134.5 eV indicate P─O bonding (Figure 1f) [21]. The gradual increase in P─O content with Li_2_S addition suggests enhanced oxygen fixation, which promotes the oxidative decomposition of Li_2_S. No sulfur species are detected in 0LS, but divalent sulfur (S^2−^) at 162.5 eV and SO_4_^2−^ components appear in 1, 3, and 5LS (Figure 1g; Figure S5) [22], consistent with increased S and Li contents (Figure S6). The analysis shows that the sample contains approximately 30% Li_2_S and 70% Li_2_SO_4_, indicating substantial partial oxidation during ball milling. To clarify the role of mechanical activation, a hand‐ground sample containing 5 wt.% Li_2_S (denoted 5LS‐M) was analyzed for comparison (Figure S7). Its XRD pattern displays sharp reflections from crystalline Li_2_S, while XPS reveals only 6% P─O bonds and no SO_4_^2−^ species. In contrast, the 5LS sample exhibits pronounced Li_2_SO_4_ formation, confirming that high‐energy ball milling effectively drives the conversion of Li_2_S into Li_2_SO_4_ and facilitates its uniform dispersion.

To gain deeper insights, the 0LS and 5LS samples were further selected for detailed comparison. Both samples exhibit secondary irregular spherical particles assembled from nanosized primary building blocks (Figure S8). Energy‐dispersive X‐ray spectroscopy (EDS) elemental mapping confirms the uniform spatial distribution of all constituent elements across the composite (Figure 1h; Figures S9 and S10). Transmission electron microscopy (TEM) and selected area electron diffraction (SAED) patterns verify the amorphous nature of both samples (Figure S11). The incorporation of Li_2_S and Li_2_SO_4_ in 5LS reduces the specific surface area while increasing the average pore size (Figure 1i; Figure S12). Although the reduced specific surface area may lower electrolyte contact to some extent, it can also help suppress parasitic side reactions and excessive electrolyte decomposition, while the enlarged pore size and reconstructed porous network preserve ion transport pathways. Pair distribution function (PDF) analysis provides atomic‐scale insight into the local bonding environment (Figure 1j). Both 0LS and 5LS are dominated by P─P and C─C correlations, indicating that the primary phosphorus‐carbon framework remains intact during modification. In contrast, 5LS exhibits additional medium‐range correlations associated with sulfur‐containing species. Specifically, a pronounced feature near ∼5.2 Å can be attributed to overlapping S─S and Li─S pair correlations consistent with Li_2_SO_4_, while a distinct signal at ∼5.72 Å corresponds to S─S correlations in Li_2_S. These signatures unambiguously confirm the coexistence of Li_2_S and Li_2_SO_4_ within the 5LS electrode. The stable presence of these dual components enables cooperative regulation of electron and ion transport and promotes the formation of a uniform and robust SEI during cycling. As a result, the 5LS electrode exhibits markedly enhanced specific capacity, rate capability, and long‐term cycling stability compared with the unmodified counterpart.

### 2.2 Electrochemical Performance

**FIGURE 2:**
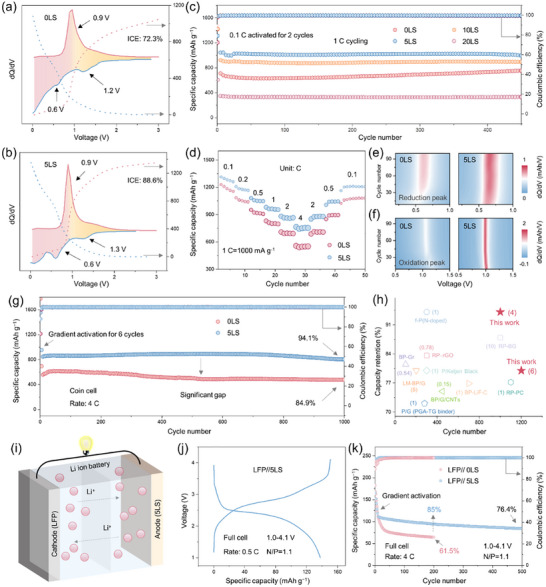
(a,b) Initial charge–discharge profiles and corresponding dQ/dV curves of the 0 and 5LS samples. (c) Long‐term cycling performance at 1 C for the 0, 5, 10, and 20LS electrodes. (d) Rate capability comparison of 0 and 5LS. (e,f) dQ/dV profiles of 0 and 5LS over 100 cycles during (e) discharge and (f) charge at 1 C. (g) Cycling stability at 4 C for the 0 and 5LS electrodes. (h) Comparison of electrochemical performance with previously reported phosphorus‐based anodes. Current densities (A g^−1^) are indicated in parentheses, and detailed references are provided in the Supporting Information. (i) Schematic diagram illustrating the configuration of the assembled full‐cell. (j) The charge and discharge curve of LFP//5LS full cell at 0.5 C. (k) Cycling stability at 4 C for the LFP//0LS and LFP//5LS full cells. The capacity is calculated based on the cathode.

A comparative analysis of RP anodes containing different Li_2_S contents was conducted to evaluate their electrochemical behavior. During the initial charge–discharge cycle, the 0, 5, 10, and 20 samples exhibited ICE of 72.3%, 88.6%, 86.2%, and 79.2%, respectively (Figure 2a,b; Figure S13). The marked improvement in ICE for 5LS indicates that a moderate amount of Li_2_S and its oxidized derivative Li_2_SO_4_ effectively promotes the conversion of RP, optimizes charge‐transport pathways, and suppresses parasitic reactions, thereby minimizing irreversible losses during the first cycle. Differential capacity (dQ/dV) profiles show a distinct reduction peak at 1.2–1.3 V, corresponding to SEI formation, followed by lithiation and delithiation peaks at 0.6–0.7 V and 0.9 V, respectively [23]. Long‐term cycling at 1 C further highlights the composition‐dependent electrochemical behavior (Figure 2c). The 0, 5, 10, and 20LS samples deliver specific capacities of 699.5, 1037.4, 920.5, and 348.0 mAh g^−1^, respectively. Notably, the optimized 5LS electrode exhibits both the highest reversible capacity and the best cycling stability, retaining 96.1% of its capacity after 450 cycles at 1 C. In contrast, the hand‐ground 5LS‐M electrode shows the poorest performance, demonstrating that high‐energy ball milling is essential for achieving an effective microstructure (Figure S14). The performance improves at moderate sulfur‑containing additives loading but deteriorates when the content exceeds 10 wt.%. This indicates that a suitable amount of sulfur‑containing additives promotes electrochemical activity, whereas excessive addition may form insulating or diffusion‐blocking regions around the phosphorus particles, thereby restricting electronic conduction and Li^+^ transport. Consequently, a fraction of the active material becomes inaccessible during cycling.

Accordingly, the 0LS and 5LS samples were selected as representative counterparts to elucidate the role of sulfur‐containing additives. Rate performance measurements reveal that 5LS consistently outperforms 0LS across all current densities, demonstrating superior rate capability and reversibility (Figure 2d). When the current density was reduced back to 0.1 C after high‐rate cycling, the 5LS anode recovered 93.5% of its initial capacity, confirming its excellent structural and interfacial integrity. dQ/dV profiles (Figure 2e,f) further indicate that 5LS maintains sharper and more intense redox peaks with minimal shift after 100 cycles, reflecting enhanced reaction kinetics, reduced polarization, and improved reversibility. Extended cycling at high rates underscores the durability of the modified electrode. At 4 C, the 5LS anode retains 94.1% of its capacity (853.4 mAh g^−1^) after 1000 cycles, greatly exceeding the 0LS electrode, which delivers 562.7 mAh g^−1^ with 84.9% retention (Figure 2g). Even at an ultrahigh rate of 6 C, 5LS maintains 794.2 mAh g^−1^ and 80% capacity retention after 1200 cycles (Figure S15). In order to figure out the synergistic mechanism of sulfur‐containing additives, 5LS without Li_2_SO_4_ control was prepared by directly mixing Li_2_S (about 1.5 wt.%) with the pre‐ball‐milled RP/C composite (Figure S16). It delivers a capacity of 671.4 mAh g^−1^ with 89% after 600 cycles, outperforming the 0LS baseline but inferior to the 5LS sample (Figure S17). These results collectively demonstrate that the outstanding electrochemical performance of 5LS originates from the synergistic effect of Li_2_S and in situ formed Li_2_SO_4_. Compared with previously reported phosphorus‐based anodes, the RP anode modified with sulfur‐containing additives exhibits markedly enhanced rate capability and stability (Figure 2h; Table S1). These results confirm the effectiveness of sulfur‐containing additives in overcoming the intrinsic kinetic and interfacial limitations of pristine RP electrodes.

To evaluate the practical feasibility of the sulfur‐containing additives modified RP anode, high‐loading electrodes were tested. As shown in Figure S18, the 5LS electrode delivers an initial capacity of 1070.7 and retains 871.7 mAh g^−1^ after 100 cycles, corresponding to a capacity retention of 81.4%. In comparison, the 0LS electrode exhibits an initial capacity of 704.1 and retains 498.3 mAh g^−1^ after 100 cycles, with a capacity retention of 70.8%. These results demonstrate that the 5LS electrode maintains superior electrochemical performance even at practically relevant loading conditions. The superior performance of the modified anode translates effectively into practical full cell configurations (Figure 2i). When paired with a lithium iron phosphate (LFP) cathode, the 5LS‐based full cell achieves a high capacity of 137.7 mAh g^−1^ at 0.5 C and maintains 110.3 mAh g^−1^ at a high rate of 4 C (Figure 2j,k; Figure S19). Its long‐term stability is equally notable, with 85% capacity retention after 200 cycles, drastically surpassing the 61.5% retention of the 0LS‐based full cell, and a remaining 76.4% after 500 cycles. This comprehensive full‐cell performance definitively confirms that the RP anode with sulfur‐containing additives is a viable candidate for fast‐charging LIBs.

### 2.3 Enhanced Conversion of Phosphorus

**FIGURE 3:**
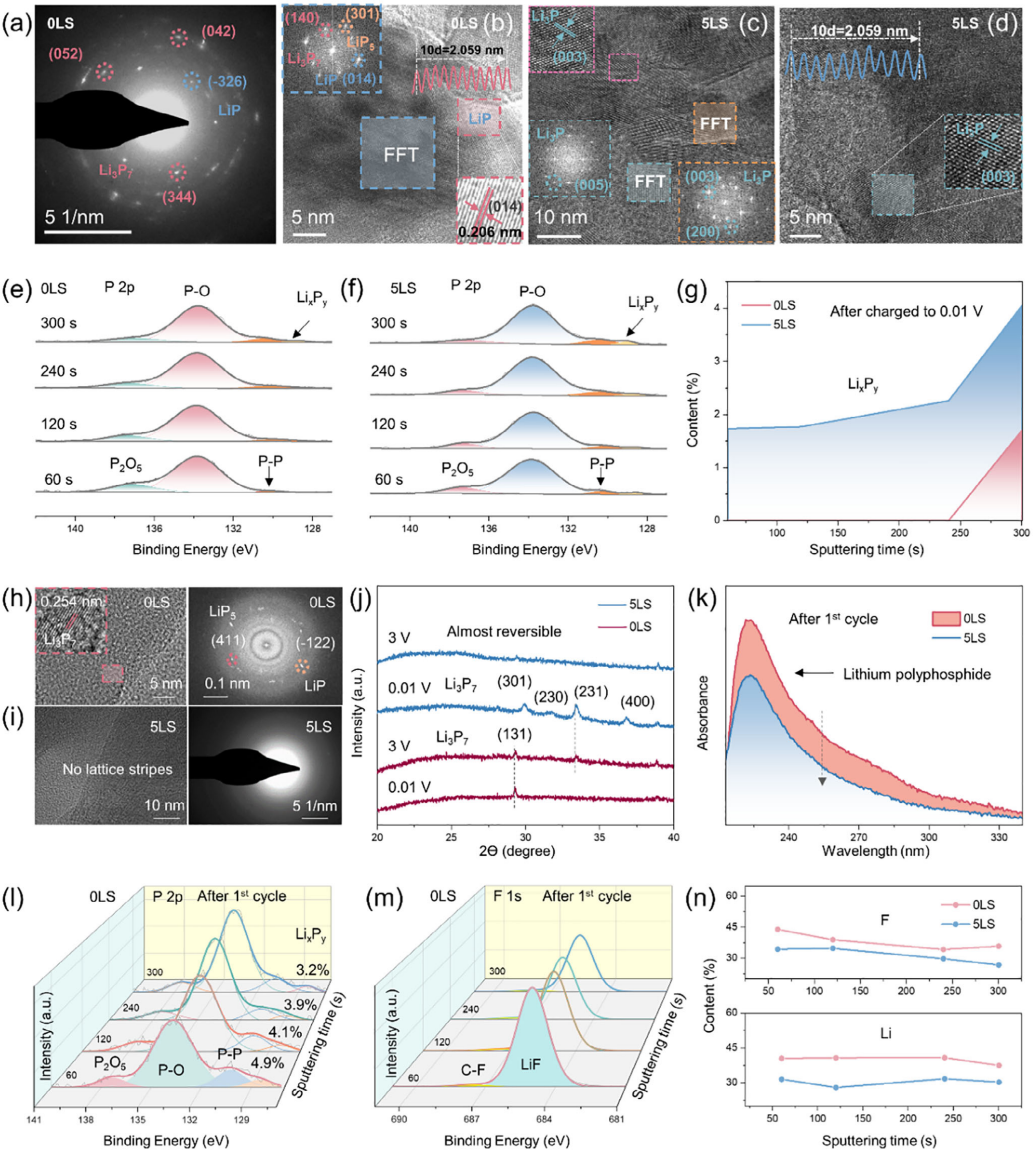
SAED pattern and TEM image of the (a,b) 0LS, (c,d) 5LS electrode after discharge to 0.01 V. (e,f) P 2p XPS spectra of 0 and 5LS electrodes after discharge to 0.01 V. (g) Quantitative comparison of Li_x_P_y_ content in 0 and 5LS after discharge to 0.01 V. (h,i) TEM images and corresponding SAED patterns of 0 and 5LS after the first cycle. (j) Ex situ XRD patterns of 0LS, (c,d) 5LS electrode. (k) UV–vis absorption spectra of 0LS and 5LS electrodes after the first cycle. (l, m) P 2p and F 1s XPS spectra of the 0LS electrode after the first cycle. (n) Comparison of F and Li contents in 0 and 5LS electrodes after the first cycle. Noted that all electrodes are conducted at 0.1 C.

The inhibited dissolution of LiPPs greatly enables a more complete electrochemical conversion of the RP anode. Direct structural evidence is provided by analyses of the fully lithiated electrodes. SAED patterns and TEM images (Figure 3a—d; Figure S20) reveal that the 0LS sample contains intermediate lithiation phases such as Li_3_P_7_, LiP, and LiP_5_ but no Li_3_P, indicating the incomplete conversion. In contrast, the fully lithiated phase Li_3_P is clearly observed in the 5LS sample, confirming a more thorough phase transformation. XPS depth profiling further supports this conclusion (Figure 3e,f). The peak at 137.1 eV corresponds to P_2_O_5_ [24], arising from surface oxidation of RP, while the peaks at 133.7, 130.3, and 128.6 eV are assigned to P─O, P─P, and lithiated phosphorus species (Li_x_P_y_, including LiP, Li_3_P_7_ and Li_3_P), respectively [25, 26]. With increasing sputtering depth, the Li_x_P_y_ peak intensity increases, signifying progressive lithiation of phosphorus into lithium phosphides. Notably, at equivalent depths, the 5LS electrode exhibits a markedly stronger Li_x_P_y_ signal than 0LS, indicating more extensive lithiation across the bulk (Figure 3g). Furthermore, ex situ XPS analysis after discharge to 0.01 V (Figure S21) reveals the persistent presence of sulfur species corresponding to Li_2_S and Li_2_SO_4_, with no obvious transformation to other sulfur chemical states observed, thereby verifying the structural and chemical stability of Li_2_S during cycling. Together, these structural and chemical analyses confirm that Li_2_S incorporation facilitates a more complete conversion reaction within the RP matrix, in excellent agreement with the accelerated reaction kinetics and superior electrochemical performance demonstrated earlier.

To assess reaction reversibility, the delithiated states of the electrodes were examined. For the 0LS sample after charging, TEM reveals lattice fringes corresponding to the intermediate phase Li_3_P_7_ (Figure 3h), and the associated SAED pattern confirms the persistence of partially converted products such as LiP and LiP_5_. In contrast, the 5LS electrode retains an amorphous structure in the delithiated state with no detectable intermediate phases (Figure 3i), indicating a highly reversible conversion reaction. XRD analysis provides further evidence (Figure 3j). After full lithiation, both 0 and 5LS display diffraction features associated with partially lithiated intermediates. However, upon subsequent delithiation, the Li_3_P_7_ phase in 5LS is almost entirely reconverted to the original amorphous state, whereas a weak Li_3_P_7_ signal remains in 0LS. These results collectively demonstrate that the incorporation of Li_2_S enables a more complete and reversible conversion process, which directly contributes to the superior cycling stability of the 5LS anode.

To further examine reaction reversibility, UV–vis absorption spectroscopy was used to detect solubleLiPPs intermediates after charging. As shown in Figure 3k, the absorption band at 222.7 nm, attributed to LiPPs, is markedly stronger for the 0LS sample than for 5LS, indicating a higher concentration of soluble intermediates and poorer reversibility in the former [27]. XPS depth profiling provides consistent evidence. In the delithiated 0LS electrode (Figure 3l), the Li_x_P_y_ signal decreases gradually with sputtering depth, suggesting the persistence of incompletely delithiated species near the subsurface region, a hallmark of sluggish reaction kinetics. In contrast, 5LS exhibits a negligible Li_x_P_y_ signal throughout the bulk (Figure S22), confirming nearly complete delithiation and excellent reversibility. Collectively, these results demonstrate that Li_2_S incorporation effectively mitigates the dissolution of LiPPs, enabling a more complete conversion reaction and markedly improved cycling reversibility.

The composition of the SEI formed after one cycle were examined by XPS. As shown in Figure 3m; Figure S23, the SEI consists of an outer organic layer, characterized by the C─F peak at 687.6 eV, and an inner inorganic‐rich layer dominated by LiF at 685.0 eV [28]. With increasing sputtering depth, the intensity of the C─F signal decreases, confirming that organic species are mainly distributed near the surface, while LiF constitutes the bulk of the inner SEI. Comparative elemental analysis (Figure 3n) reveals that the 0LS electrode contains higher F and Li contents than 5LS. The elevated F concentration indicates a thicker, LiF‐rich, and mechanically fragile SEI, whereas the increased Li signal suggests the presence of residual lithium compounds resulting from incomplete delithiation. Electrochemical impedance spectroscopy (EIS) further supports these findings (Figure S24). The fitted R_ct_ of 5LS is 30.2 Ω, less than half that of 0LS, indicating substantially enhanced interfacial charge‐transfer kinetics [29]. These improvements can be attributed to the effect of Li_2_SO_4_, which promotes the formation of a uniform, inorganic‐rich, and mechanically adaptive SEI that effectively suppresses parasitic reactions and enhances interfacial stability.

### 2.4 Reaction Kinetics Properties

**FIGURE 4:**
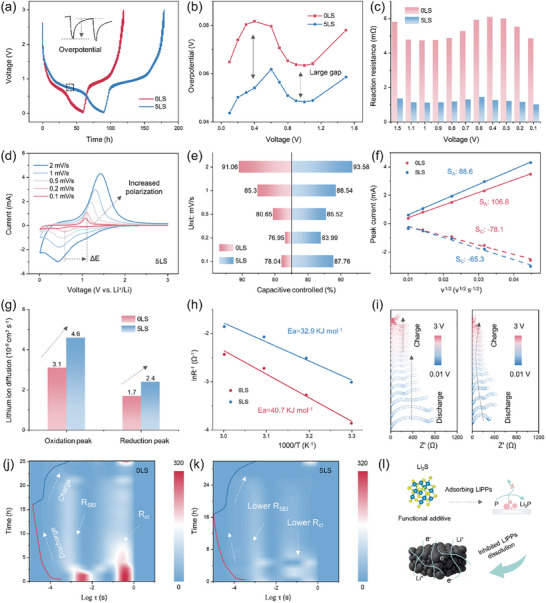
(a) GITT profiles of the 0 and 5LS samples. The inset shows an enlarged view of the highlighted region. (b) Overpotential evolution of 0 and 5LS during discharge derived from GITT curves. (c) Reaction resistances of 0 and 5LS extracted from GITT measurements. (d) CV curves of the 5LS electrode at various scan rates. (e) Capacitive contribution ratios of the 0 and 5LS electrodes at different scan rates. (f) Fitting plots of current (I) vs. the square root of scan rate (v^1/2^) at the reductive and oxidative peak potentials. (g) Calculated Li^+^ diffusion coefficients of 0 and 5LS. (h) Apparent desolvation activation energy (E_a_) of Li^+^ ions for 0 and 5LS. (i) In situ EIS profiles recorded during charge‐discharge cycling. (j,k) Distribution of relaxation time (DRT) maps for 0 and 5LS; insets show corresponding charge‐discharge profiles. (l) Schematic illustration of the improved reaction kinetics enabled by sulfur‐containing compounds modification.

The more complete electrochemical conversion of the RP anode can greatly promote the reaction kinetics, which was systematically examined. Galvanostatic intermittent titration technique (GITT) measurements were first performed to assess electrode polarization and kinetic behavior (Figure 4a). The 5LS electrode exhibits a markedly lower overpotential than 0LS throughout the discharge process (Figure 4b), indicating reduced polarization and more facile Li^+^ insertion and extraction, both essential for fast reaction kinetics [30]. Correspondingly, the total reaction resistance, comprising ion transport and charge‐transfer, remains substantially lower in 5LS across all states of charge (Figure 4c), suggesting fewer kinetic barriers and more efficient Li^+^ transport [31]. Further insights were obtained from cyclic voltammetry (CV) at scan rates ranging from 0.1 to 2 mV s^−1^ (Figure 4d; Figure S25). With increasing scan rate, both anodic and cathodic peaks shift gradually, accompanied by a proportional increase in current. Across this range, 5LS consistently displays a smaller peak separation (ΔE) than 0LS (Figure S26), reflecting enhanced reversibility and diminished polarization [2, 32]. Quantitative analysis of the current response reveals that 5LS possesses a significantly higher capacitive contribution at all scan rates (Figure 4e; Figure S27), highlighting its surface‐dominated and rapid kinetic behavior [33]. The Li^+^ diffusion characteristics were further quantified from CV profiles. As shown in Figure 4f, the 5LS anode exhibits a steeper slope during delithiation and a shallower slope during lithiation relative to 0LS, indicating accelerated Li^+^ diffusion and insertion kinetics [34]. The calculated diffusion coefficients (Figure 4g) confirm these observations, collectively demonstrating that Li_2_S incorporation establishes efficient ion‐transport pathways and substantially enhances the overall electrode kinetics.

The interfacial kinetics and charge transport behavior were further examined using activation energy analysis and in situ impedance spectroscopy. The activation energy (E_a_) for Li^+^ desolvat ion, which represents the energy barrier for ion transfer across the electrode‐electrolyte interface, is markedly lower for the 5LS electrode than for 0LS (Figure 4h; Figure S28). This reduced E_a_ indicates a more facile desolvation process, thereby accelerating interfacial Li^+^ transport [35]. These kinetic benefits are further supported by in situ EIS, which monitors the evolution of interfacial resistance during cycling (Figure 4i–k). The results reveal that both the solid‐electrolyte interphase (R_SEI_, high frequency) and charge transfer (R_ct_, medium frequency) progressively decrease during cycling, with the 5LS sample showing a much more pronounced reduction [36]. This behavior suggests that the initially loose and heterogeneous SEI undergoes continuous reconstruction into a denser and more conductive interphase during cycling [37]. In the early stage, the SEI is rich in organic components and partially decomposed electrolyte species, which contribute to high interfacial resistance. During cycling, these unstable organic components further decompose and rearrange into more stable inorganic species, thereby lowering ion‐transport resistance.

In summary, Li_2_S functions as a confinement additive by first suppressing the dissolution and diffusion of LiPPs intermediates. By confining these intermediates at the reaction interface, it subsequently enables a more complete and reversible conversion reaction toward the final Li_3_P phase (Figure 4l). Meanwhile, the in situ generated Li_2_SO_4_ selectively absorbs solvent molecules and guides the formation of a uniform, Li_2_O‐dominated interphase.

### 2.5 Interface Properties

**FIGURE 5:**
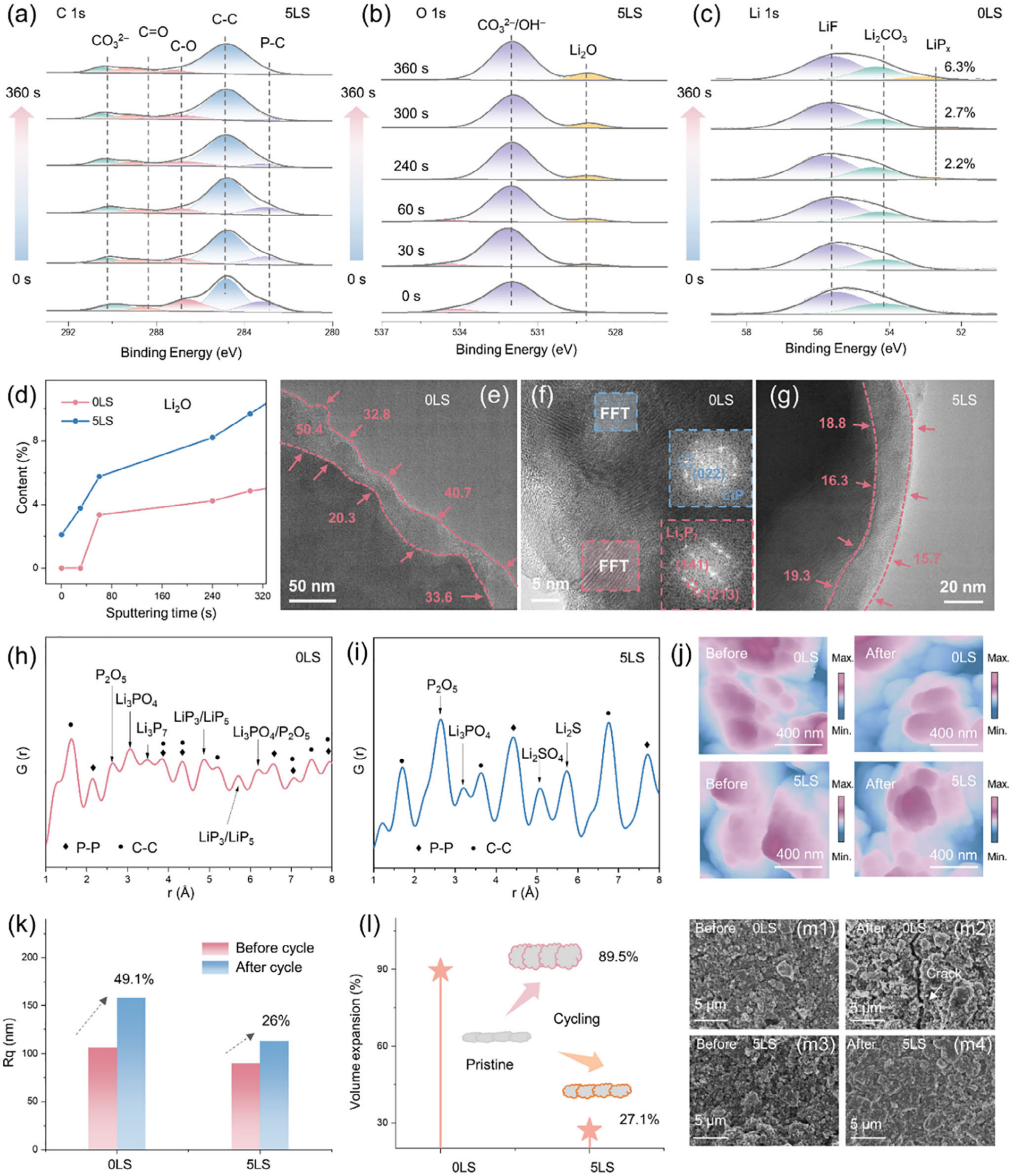
(a,b) C 1s and O 1s spectra of 5LS after 100 cycles. (c) Li 1s spectra of 0LS after 100 cycles. (d) Relative Li_2_O content of 0 and 5LS derived from O 1s spectra after 100 cycles. TEM images of (e,f) 0LS, (g) 5LS after 100 cycles. (h,i) PDF data of 0 and 5LS after 100 cycles. The diamond symbol denotes P─P bonds and the dot symbol denotes C─C bonds. (j) AFM images of 0 and 5LS before and after 100 cycles. (k) Surface roughness (R_q_) of 0 and 5LS before and after 100 cycles. (l) Volume expansion of 0LS and 5LS electrodes after 100 cycles. (m) Electrode morphology of 0 and 5LS before and after 100 cycles. Noted that all electrodes are conducted at 4 C.

To assess long‐term interfacial stability, the SEI formed after 100 cycles at 4 C was thoroughly characterized. XPS analysis was first conducted to confirm the stability of sulfur‑containing additives after 100 cycles. The S 2p XPS depth profiles show persistent SO_4_^2^^−^ and S^2^^−^ signals at all sputtering times, indicating Li_2_S and Li_2_SO_4_ exist stably in both the surface and bulk of the electrode (Figure S29). Further XPS analysis was then performed to determine the chemical composition of the SEI layer. As shown in Figure 5a; Figures S30 and S31, the C 1s spectra exhibit peaks at 283.2, 284.8, 286.7, 288.6, and 290.1 eV, corresponding to P─C, C─C, C─O, C═O, and CO_3_^2−^ species, respectively [38, 39, 40]. Notably, the 5LS electrode demonstrates the largest content of Li_2_CO_3_ than 0 and 5LS without Li_2_SO_4_ electrodes, and this highest content is accompanied by a relatively even distribution of these species through the SEI (Figure S32). The P─C bonds, formed via electrolyte decomposition, are essential for maintaining electronic conductivity and structural integrity. As illustrated in Figure S33, the 5LS electrode shows a higher surface concentration of P─C species with a steep compositional gradient along the depth, indicating a stable surface interface that facilitates charge transport and mitigates cyclic degradation. The O 1s spectra (Figure 5b; Figures S34 and S35) display a characteristic peak at 529.1 eV associated with Li_2_O [41]. The continuous increase in Li_2_O signal intensity with sputtering depth (Figure 5d; Figure S36) confirms its enrichment in the inner SEI region, with the greatest amount of Li_2_O in the 5LS sample compared with the 0LS and 5LS without Li_2_SO_4_ sample. This comparison suggests that Li_2_SO_4_ plays an important role in promoting the formation of a robust and ion‐conductive inorganic‐rich SEI. In contrast, F‐content analysis (Figure S37) reveals that 0LS contains more LiF than 5LS, consistent with the first‐cycle XPS results. These findings indicate that the SEI of 5LS is enriched with P─C species on the surface and dominated by Li_2_O in the bulk, with a limited amount of brittle LiF. Moreover, LiP_x_ components, which are signatures of irreversible reactions, are detected only in the 0LS electrode (Figure 5c; Figures S38 and S39), further confirming its higher susceptibility to parasitic side reactions [42, 43]. The greater P content observed in 0LS (Figure S40) provides additional evidence of incomplete reversibility. Overall, the 5LS electrode develops a stable SEI featuring a P─C─rich surface and an inorganic‐rich inner matrix dominated by Li_2_O with minimal LiF. This gradient architecture accommodates the large volume fluctuations of RP anodes, effectively suppressing electrolyte decomposition and ensuring interfacial stability during prolonged cycling. Both theoretical calculations and experimental characterization confirm the role of Li_2_SO_4_ in facilitating the formation of a stable SEI.

To investigate the interfacial and structural evolution after extended cycling, a combination of TEM, PDF, and atomic force microscopy (AFM) analyses was conducted. As shown in Figure 5e, the 0LS electrode is coated with a heterogeneous SEI layer exhibiting a variable thickness of 20.3–50.4 nm, which facilitates electrolyte infiltration and promotes continuous parasitic decomposition. High‐resolution TEM (Figure 5f) reveals crystalline LiP and Li_3_P_7_ phases in 0LS, providing direct evidence of incomplete reversibility and undesired side reactions. In contrast, the 5LS electrode displays a uniform and compact SEI with a narrower thickness range of 15.7–19.3 nm (Figure 5g), indicative of suppressed interfacial degradation. PDF analysis was employed to probe local structural evolution after 100 cycles (Figure 5h,i). The P─P and C─C correlations confirm that the primary framework of both 0LS and 5LS remains intact, while lithium phosphate species detected in both samples arise from side reactions during cycling. Notably, intermediate lithiation products such as Li_3_P_7_, LiP_3_, and LiP_5_ are evident in 0LS but absent in 5LS, underscoring a more direct and reversible conversion pathway in the modified electrode. Persistent detection of Li_2_S and Li_2_SO_4_ in 5LS after prolonged cycling further confirms its electrochemical stability as regulators of reaction kinetics and interfacial chemistry.

The mechanical stability of the SEI was evaluated by AFM. As shown in Figure S41, the 5LS sample exhibits a lower mean Young's modulus than 0LS, indicating a more compliant interphase. Rather than suggesting a stiffer or intrinsically stronger SEI, this lower modulus implies that the interphase formed on the modified electrode is more deformable and therefore better able to accommodate the repeated volume changes during cycling [44]. Consistent with this interpretation, after 100 cycles the surface roughness of 0LS increases by 49.1%, whereas that of 5LS increases by only 26% (Figure 5j,k). This pronounced difference suggests that the SEI on 0LS undergoes repeated fracture and reformation, while the more stable interphase on 5LS remains intact and can better buffer volume strain [45]. As a result, the 5LS electrode exhibits limited volume expansion of 27.1%, markedly lower than the 89.5% observed in 0LS (Figure 5l; Figure S42). Scanning electron microscopy (SEM) images (Figure 5m) further confirm the preserved electrode morphology in 5LS, in contrast to severe cracking observed in 0LS after extended cycling [46]. Collectively, these multimodal post‐cycling analyses demonstrate that the Li_2_SO_4_ in the 5LS electrode synergistically contributes to a stable and mechanically resilient SEI, which effectively suppresses parasitic reactions, mitigates volume strain, and maintains electrode integrity, thereby ensuring long‐term cycling stability.

## 3 Conclusion

In summary, we establish a synergistic strategy that couples reaction‐kinetics regulation with interfacial stabilization to overcome the intrinsic limitations of RP anodes. Li_2_S suppresses LiPPs dissolution and promotes reversible conversion, while Li_2_SO_4_ directs the formation of a robust inorganic interphase that accommodates large volume changes. This cooperative modulation enables high efficiency, fast kinetics, and exceptional cycling stability across wide rate regimes, including sustained operation at high current densities. Beyond delivering competitive electrochemical performance, this work provides a generalizable design framework for stabilizing conversion‐type anodes and advancing their practical implementation in high‐energy lithium‐ion batteries.

## Conflicts of Interest

The authors declare no conflicts of interest.

## Supporting information




**Supporting File**: advs75457‐sup‐0001‐SuppMat.docx.

## Data Availability

The data that supports the findings of this study are available in the supplementary material of this article.
