# Supplementary material for: Sulfur‑Containing Additives for Enhanced Kinetics and Interfacial Stability of Phosphorus Anodes in Li‑Ion Batteries
Source: Adv Sci (Weinh). 2026 May 25:e75457. Online ahead of print. doi: 10.1002/advs.75457 (PMC13335658; doi:10.1002/advs.75457)
Supplement: Supplementary file 1 [file ADVS-9999-e75457-s001.docx]

Supporting Information 

**Sulfur‑Containing Additives for Enhanced Kinetics and Interfacial Stability of Phosphorus Anodes in Li‑Ion Batteries**

Huixian Xie, Xinze Li, Zhenjiang Yu, Hongyi Chen, Lingwen Liu and Kwun Nam Hui*

H. Xie, X. Ze, H. Chen, L. Liu, Prof. K. N. Hui

Joint Key Laboratory of the Ministry of Education, Institute of Applied Physics and Materials Engineering, University of Macau, Avenida da Universidade, Taipa, Macau SAR, 999078, P.R. China

E-mail: bizhui@um.edu.mo

Dr. Z. Yu

Department of Chemistry, Lancaster University, Lancaster LA1 4YB, United Kingdom

Huixian Xie, Xinze Li, Zhenjiang Yu contributed equally to this work

**Material preparation**

**Synthesis of RP-based composites**

The control sample (denoted as 0LS) was prepared by ball-milling a stoichiometric mixture of red phosphorus (RP, 40 wt.%, Aladdin, 99.99%) and carbon (Super P, 60 wt.%, Canrd Technology Co. Ltd.) under an argon atmosphere. The milling process was conducted in stainless steel jars using 10 mm and 5 mm diameter steel balls in a 1:1 quantity ratio, with a ball-to-powder mass ratio of 70:1. Milling was performed at 800 rpm for 6 hours.

For the optimized samples, lithium sulfide (Li_2_S, Aladdin, AR) was introduced at varying concentrations of 1, 3, 5, 10, and 20 wt.%, while the RP content was fixed at 40 wt.%. The carbon content was adjusted accordingly to 59, 57, 55, 50, and 40 wt.%, respectively. The resulting samples were labeled as 1LS, 3LS, 5LS, 10LS, and 20LS. The 0LS-M and 5LS-M control samples were prepared by direct manual grinding of the precursors in an argon-filled glove box, without any high-energy ball milling treatment. The control sample denoted as 5LS without Li_2_SO_4_ was prepared by directly mixing Li_2_S (about 1.5 wt.%) with the pre-ball-milled RP/C composite, followed by manual grinding in an argon-filled glove box.

**Electrochemical measurements**

The electrode slurry was prepared by homogenizing the active material, acetylene black, and polyvinylidene fluoride (PVDF) binder at a mass ratio of 7.0:1.5:1.5 in N-methyl-2-pyrrolidone (NMP) solvent via magnetic stirring. The resulting homogeneous slurry was then coated onto a copper foil current collector and pre-dried at 60 °C. After 10 minutes, the electrode was transferred to a vacuum drying oven and further dried at 60 °C for 8 hours. The dried electrode was then calendared and punched into 10-mm-diameter discs for cell assembly. Unless otherwise noted, the mass loading of the active material is 0.7-0.8 mg cm^-2^. Coin-type half-cells (CR2032) were assembled in an argon-filled glove box (Lab2000, Etelux). Lithium metal foil and a polypropylene (PP) separator were used as the counter electrode and separator, respectively. The electrolyte was 1 M LiPF_6_ in a mixture of ethylene carbonate (EC) and diethyl carbonate (DEC) (1:1 by volume) containing 5 wt.% fluoroethylene carbonate (FEC), all purchased from Canrd Technology Co. Ltd. The electrochemical performance of the cells was evaluated using a Neware CT-4000 battery testing system (Shenzhen, China) within a voltage window of 0.01-3.0 V. The specific capacity is calculated based on the composite. 1 C rate is 1000 mA g^-1^. To clarify, the reported percentage capacity values at different current densities are calculated relative to the initial capacity at each corresponding rate, rather than being normalized to the 1 C capacity.

The full cell was assembled using LiFePO_4_ (LFP) as the cathode. The cathode was fabricated by blending LiFePO_4_, acetylene black, and PVDF binder at a weight ratio of 8:1:1 in NMP. The slurry was cast onto a current collector and dried at 120 ^o^C for 8 hours under vacuum, following a procedure analogous to that of the anode preparation. The electrolyte was 1 M LiPF_6_ in a mixture of ethylene carbonate (EC) and diethyl carbonate (DEC) (1:1 by volume). For the LFP//5LS full cell, the N/P ratio was controlled about 1.1, and the cell was tested within a voltage window of 1.0-4.1 V. The capacity is calculated based on the cathode and the 1 C is 150 mA g^-1^.

Galvanostatic intermittent titration technique (GITT) measurements were performed by applying a 0.1 C current pulse for 10 min, followed by a 40 min open-circuit rest. Cyclic voltammetry (CV), electrochemical impedance spectroscopy (EIS), and in-situ EIS were conducted using an electrochemical workstation (VMP-300, Bio-Logic, Germany). The frequency range for impedance measurements was set from 100 kHz to 0.1 Hz.

This rapid kinetics is confirmed by the relationship between the current response and the applied scan rate, which follows the equation:^[1]^

I(v)=k_1_ v+k_2_ v^1/2^                                                                                                          (1)

The current response can be deconvoluted into two distinct components: a surface-capacitive process (k_1 _ν) and a diffusion-limited process (k_2_ ν^1/2^).^[2]^ By calculating the parameters k_1_ and k_2_ at fixed potentials, the individual contribution of each process can be precisely determined.

The ion diffusion coefficient, deemed a critical factor in determining electrochemical reaction rates, can be calculated by the following well-established equation:^[3]^

I_p_ = 2.69 $×$ 10^5^n^3/2^AD^1/2^v^1/2^C_Li+_                                                                                           (2)

where, I_p_, n, A, D, v, and C_Li+_ are the peak current, the transform electrons in the reaction, the electrode area, the ions diffusion coefficient, the scan rates, and the concentration of lithium ions, correspondingly.

According to the Arrhenius equation as following^[4]^:

$\frac{1}{Rct}=Aexp\frac{-Ea}{RT}$                                                                                                            (3)

In the equation, R_ct_, A, E_a_, R, and T are the charge-transfer resistance, Arrhenius constant, desolvated activation energy, gas constant, and absolute temperature, respectively.

**Measurement of electrode density and porosity**

Electrode density and porosity are calculated on the high loading electrode. 

The tap density (ρ_tap_) is calculated based on the following formula:

$ρ_{tap}=\frac{M_{coating}}{A×T_{coating}}$

where M_coating_ is the electrode mass after subtracting the current collector, T_coating_ is electrode thickness after subtracting the current collector.

For the 8 mm-diameter electrode disks:

A=0.5026 cm^-2^

M_coating_=0.00139 g

T_coating_=0.0023 cm

Therefore, both 0LS and 5LS electrodes exhibit nearly identical compaction density of ~1.2 g cm^-3^.

The total true density (ρ_true_) of the composite is calculated based on the following formula:

$ρ_{true}=\frac{1}{∑(\frac{w_{i}}{ρ_{i}})}$

where $w_{i}$ is the mass fraction of the component $i$, $ρ_{i}$ is the intrinsic true density of the component $i$.

Intrinsic true densities of component: $ρ_{PVDF}$=1.77 g cm^-3^, $ρ_{RP}$=2.2 g cm^-3^, $ρ_{sp}$=2 g cm^-3^, $ρ_{li2S}$=1.66 g cm^-3^, $ρ_{li2SO4}$=2.22 g cm^-3^

Mass fractions of 0LS electrode: $w_{PVDF}$= 0.15306, $w_{RP}$=0.27755, $w_{SP}$=0.56938

Mass fractions of 5LS electrode: $w_{PVDF}$=0.15306, $w_{RP}$=0.27755, $w_{SP}$=0.53469, $w_{Li2s}$=0.0104, $w_{Li2SO4}$=0.0243

True density of 0LS and 5LS electrode is 2.0108 g cm^-3^ and 2.0133 g cm^-3^

The electrode porosity (ε) is calculated based on the following formula:

$ε=(1-\frac{ρ_{tap}}{ρ_{true}})×100\%$

The electrode porosity of 0LS and 5LS electrode is 40.21% and 40.29%.

0LS and 5LS electrodes exhibit nearly identical electrode density (~1.2 g cm^-3^) and total porosity (~40%), with negligible differences between samples.

**Material characterization**

The crystal structure of the samples was characterized by X-ray diffraction (XRD) on a Rigaku Smartlab 9000 W diffractometer with Cu Kα radiation (λ = 1.54181 Å). Morphological and microstructural analyses were performed using a Zeiss Sigma field emission scanning electron microscope (FE-SEM) and a JEM-F200 transmission electron microscope (TEM). Elemental distribution was analyzed by energy-dispersive X-ray spectroscopy (EDS) mapping on a JEOL JEM-2100 microscope operating at 200 kV. The specific surface area was determined from nitrogen adsorption isotherms using the Brunauer-Emmett-Teller (BET) method on a Micromeritics surface characterization analyzer. Chemical states were probed by X-ray photoelectron spectroscopy (XPS) on a Thermo Fisher Scientific instrument with an Al Kα monochromatic source (1486 eV). UV-visible spectra were acquired using a Jasco V-770 spectrophotometer, and surface topography was examined by atomic force microscopy (AFM) on a Bruker FastScanBio Icon system. Pair distribution function (PDF) analysis was carried out using total scattering data collected in-house on a Rigaku Oxford Diffraction SuperNova single-crystal diffractometer equipped with a Mo source (λ = 0.07107 nm) and an ATLAS-S2 CCD area detector, providing a Q_max_ of 14-16 Å^-1^. Data were normalized with PDFgetX2^1^ and instrumental resolution effects were corrected by measuring a LaB_6_ standard.

**Computational Methods**

Spin-polarized density functional theory (DFT) calculations were performed using the Vienna *ab initio* simulation package (VASP).^[5]^ The electron–ion interactions were described by the projector augmented-wave (PAW) method with a plane-wave basis set and a kinetic energy cutoff of 450 eV. The exchange–correlation functional was treated within the generalized gradient approximation (GGA) using the Perdew–Burke–Ernzerhof (PBE) parametrization.^[6]^ The Brillouin zone was sampled with a Γ-centered 2×2×1 k-point mesh. The convergence criteria for electronic self-consistent field (SCF) iterations and ionic relaxation steps were set to 10⁻⁵ eV and 0.05 eV·Å^-^¹, respectively. Spin-polarized calculations were considered where necessary.

Lattice parameters of structure were a = 16.40 Å, b = 9.84 Å, and c = 16.78 Å. The amorphous structure was generated using the Bond‑Switching Monte Carlo method. A symmetric slab model with a thickness corresponding to three stoichiometric layers was constructed to represent the surface. To eliminate spurious interactions between periodic images along the direction perpendicular to the surface, a vacuum layer of 20 Å was introduced. During geometry optimization of the surface systems, the bottom two atomic layers were held fixed, while all other atoms were allowed to relax fully. Van der Waals interactions were included via Grimme’s DFT-D3 dispersion correction.^[7]^ The adsorption energy ($E_{\text{ads}}$) was calculated using the following equation:

E_ads_ = E_ad/sub_-E_ad_-E_sub_

where E_ad/sub_, E_ad_, and E_sub_ are the total energies of the optimized adsorbate/substrate system, the adsorbate in the gas phase, and the clean substrate, respectively.

**Figure S1.** (a) O 1s and (b) P 2p spectra of pristine RP. (c) O 1s spectrum of conductive carbon.

**Figure S2.** O 1s spectra of (a) 0LS-M and (b) 5LS-M. (c) P 2p spectra of 0LS-M.

**Figure S3**. O 1s spectra of 0LS and 5LS.

**Figure S4.** Charge density diﬀerence maps showing the interfacial electron redistribution between DEC and Li_2_SO_4_ surface (Purple: electron accumulation, pink: electron depletion).

**Figure S5**. S 2p XPS spectra of 5LS electrodes from different batches, demonstrating high reproducibility of the electrode composition.

**Figure S6.** Li 1s spectra of the samples with different addition amounts of lithium sulfide.

**Figure S7**. XRD pattern, P 2p and S 2p spectrum of the hand ground sample.

**Figure S8.** SEM image of (a) 0LS and (b) 5LS samples.

**Figure S9.** EDS mapping images of the 0LS sample.

**Figure S10**. TEM-EDS mapping of 5LS sample.

**Figure S11.** TEM images and corresponding SAED patterns of (a-c) 0LS and (d-f) 5LS samples.

**Figure S12**. (a) Nitrogen adsorption-desorption isotherms and (b) pore size distribution of 

0LS and 5LS respectively. 

**Figure S13**. Initial charge-discharge profiles of the 10LS, and 20LS samples.

**Figure S14**. Long cycle performance at 1C of 1LS and 5LS-M sample.

**Figure S15**. Long cycle performance at 6C of 0LS and 5LS samples.

**Figure S16**. S 2p spectrum of 5LS without Li_2_SO_4_ sample.

**Figure S17**. Long cycle performance at 4C of 5LS without Li_2_SO_4_ sample. After 600 cycles, the 0LS and the Li_2_S-only control (5LS without Li_2_SO_4_) retain 86.6% and 89% of their initial capacities, respectively, whereas the 5LS electrode achieves nearly 100% capacity retention.

**Figure S18**. Cycling stability at 1 C for the 0LS and 5LS high loading electrodes. 1C=1000 mAh g^-1^, and the mass loading is about 3 mg cm^-2^.

**Figure S19**. Cycling performance of the LFP// 5LS full cells with different N/P ratios at 4 C. N/P value of 1.1 provided the best balance between capacity matching and cycling stability. Therefore, an N/P ratio of 1.1 is considered to be the optimal compromise for the full-cell configuration in this study.

**Figure S20**. TEM image of 0LS sample after discharged to 0.01 V.

**Figure S21**. Depth-dependent S 2p XPS spectra of the 5LS electrode after discharge to 0.01 V.

**Figure S22**. P 2p spectrum of 5LS sample after 1st cycle.

**Figure S23**. F 1s spectrum of 5LS sample after 1st cycle.

**Figure S24**. EIS curves of 0LS and 5LS after 1st cycle.

**Figure S25**. CV curves of the 0LS at various scan rates.

**Figure S26**. Peak potential difference (ΔE) between oxidation peak and reduction peak at various scan rates.

**Figure S27**. The percentage of pseudo capacitance in (a) 0LS and (b) 5LS samples at 2 mV s^-1^.

**Figure S28**. EIS curves of (a) 0LS and (b) 5LS samples at various temperatures after being one cycle activation.

**Figure S29**. Depth-dependent S 2p XPS spectra of the 5LS electrode after 100 cycles.

**Figure S30**. C 1s spectrum of 0LS after 100 cycles.

**Figure S31**. C 1s spectrum of 5LS without Li_2_SO_4_ after 100 cycles.

**Figure S32**. Li_2_CO_3_ content of 0LS, 5LS and 5LS without Li_2_SO_4_ after 100 cycles.

**Figure S33**. P-C content of 0LS and 5LS after 100 cycles.

**Figure S34**. O 1s spectra of 0LS after 100 cycles.

**Figure S35**. O 1s spectrum of 5LS without Li_2_SO_4_ after 100 cycles.

**Figure S36**. Li_2_O content of 5LS without Li_2_SO_4_ after 100 cycles.

**Figure S37**. F content of 0LS and 5LS after 100 cycles.

**Figure S38**. Li 1s spectra of 5LS after 100 cycles.

**Figure S39**. P 2p XPS spectra of 0LS and 5LS electrodes after 100 cycles.

**Figure S40**. P content of 0LS and 5LS after 100 cycles.

**Figure S41.** AFM Young’s modulus maps of the SEI layer on (a) 0LS and (b) 5LS electrodes after 100 cycles.

**Figure S42**. SEM cross section of 0LS and 5LS samples before and after 100 cycles. The 0LS electrode exhibited a thickness of 16.2 ± 0.2 μm (n = 3) after cycling, and the 5LS electrode exhibited a thickness of 10.4 ± 0.5 μm (n = 3).

**Supplementary Table 1**: Comparison of electrochemical performance with other pervious works about P-based anodes for LIBs. Note that the specific capacity and current density are calculated based on the total weight of P/C composite. The main text presents a comparison with only the first 10 works.

| Materials | P mass loading | Current density (A/g) | Capacity (mAh/g) | Cycle number | ICE | Retention | Reference |
| --- | --- | --- | --- | --- | --- | --- | --- |
| 5LS | 40% | 4 | 2133.4 | 1000 | 88.6% | 94.1% | This work |
|  |  | 6 | 1800.7 | 1200 |  | 80% |  |
| f-P(N-doped) | 100% | 1 | 1221 | 300 | - | 94.1% | ^[8]^ |
| RP-BG | 60% | 10 | 531.5 | 1000 | 84.6% | 87.9% | ^[9]^ |
| RP–rGO | 57.1% | 0.78 | 1650 | 300 | 82% | 83.6% | ^[10]^ |
| FP-G | 50% | 4 | 1259.6 | 1300 | 79.9% | 83.2% | ^[11]^ |
| BP-Graphite | 30.3% | 0.52 | 2270 | 100 | 85.5% | 81.5% | ^[12]^ |
| P/Ketjen Black | 70% | 1 | 1359.3 | 300 | 76.4% | 80% | ^[13]^ |
| LM-BP/G | 70% | 5 | 1403.8 | 200 | 97% | 80% | ^[14]^ |
| RP-PC | 49.4% | 1 | 1250 | 1100 | 70.4% | 77.2% | ^[15]^ |
| BP-LiF-C | 60% | 1 | 1204.3 | 700 | 86.8% | 76.9% | ^[16]^ |
| BP/G/CNTs | 50% | 0.15 | 1375 | 450 | 71.45% | 75% | ^[17]^ |
| P/G (PGA-TG binder) | 70% | 1 | 2000 | 280 | 86.84% | 72.1% | ^[18]^ |
| RP-CNT | 72% | 1 | 1206 | 450 | 67% | 72% | ^[19]^ |
| RP Nanospheres | 60% | 1 | 1500 | 600 | 80.1% | 69.8% | ^[20]^ |
| P-CoPPc/GO | 70% | 0.2 | 832 | 800 | - | 68.8% | ^[21]^ |
| P-graphite | 70% | 0.25 | 2200 | 200 | 76% | 68.4% | ^[22]^ |
| RP-sulfur/carbon | 81% | 2 | 750 | 450 | 69.5% | 63.2% | ^[23]^ |
| P/ZIF-8 | 30% | 0.1 | 1603 | 100 | 76.7% | 49% | ^[24]^ |
| BP@CNTs Hybrid | 68.92% | 0.5 | 1600 | 650 | 77.4% | 32.6% | ^[25]^ |

**References**

[1]	W. Xiao, X. Li, B. Cao, G. Huang, C. Xie, J. Qin, H. Yang, J. Wang, X. Sun, *Nano Energy* **2021**, *83*, 105772.

[2]	X. Yang, R.-Y. Zhang, J. Zhao, Z.-X. Wei, D.-X. Wang, X.-F. Bie, Y. Gao, J. Wang, F. Du, G. Chen, *Adv. Energy Mater.* ***2018****, 8, 1701827*.

[3]	P. Jing, Q. Wang, B. Wang, X. Gao, Y. Zhang, H. Wu, *Carbon* **2020**, *159*, 366.

[4]	S. Zhang, M. Ye, Y. Zhang, Y. Tang, X. Liu, C. C. Li, *Adv. Funct. Mater.* ***2023****, 33 2208230.*

[5]	P. Qiu, Y. Yao, W. Li, Y. Sun, Z. Jiang, B. Mei, L. Gu, Q. Zhang, T. Shang, X. Yu, J. Yang, Y. Fang, G. Zhu, Z. Zhang, X. Zhu, T. Zhao, W. Jiang, Y. Fan, L. Wang, B. Ma, L. Liu, Y. Yu, W. Luo, *Nano Lett.* **2021**, *21*, 700.

[6]	G. Zhu, Y. Jiang, H. Yang, H. Wang, Y. Fang, L. Wang, M. Xie, P. Qiu, W. Luo, *Adv. Mater.* **2022**, *34*, e2110128.

[7]	M. Fang, J. Han, S. He, J. C. Ren, S. Li, W. Liu, *J. Am. Chem. Soc.* **2023**, *145*, 12601.

[8]	Z. Duan, X. Feng, G. Lai, D. Liu, X. Zhang, H. Wang, S. Chen, X. He, Z. Liu, L. Tong, H. Wang, X. F. Yu, J. Wang, *J. Colloid. Interface Sci.* **2025**, *679*, 161.

[9]	C. Ji, Q. Zhou, Y. Yuan, W. Chen, T. Hou, B. Zhao, Y. Qin, X. Xiong, *Energy & Environ. Sci.* ***2024****, 17, 4273*.

[10]	T. Wang, F. Cheng, N. Zhang, W. Tian, J. Zhou, R. Zhang, J. Cao, M. Luo, N. Li, L. Jiang, D. Li, Y. Li, K. Liang, H. Liu, P. Chen, B. Kong, *Adv. Eng. Mater.* **2021**, *23*, 2001507.

[11]	X. Gao, L. Liu, L. Zu, H. Lian, X. Cui, X. Wang, *J. Power Sources* **2024**, *595,* 234077.

[12]	J. Sun, G. Zheng, H. W. Lee, N. Liu, H. Wang, H. Yao, W. Yang, Y. Cui, *Nano Lett.* **2014**, *14*, 4573.

[13]	X. Li, G. Chen, Z. Le, X. Li, P. Nie, X. Liu, P. Xu, H. B. Wu, Z. Liu, Y. Lu, *Nano Energy* **2019**, *59*, 464.

[14]	S. Zhang, Y. Wan, Y. Cao, Y. Zhang, H. Gong, X. Liang, B. Zhang, X. Wang, S. Fang, J. Wang, W. Li, J. Sun, *eScience* **2025**, *5*, 100328.

[15]	S. Zhang, C. Liu, H. Wang, H. Wang, J. Sun, Y. Zhang, X. Han, Y. Cao, S. Liu, J. Sun, *ACS Nano* **2021**, *15*, 3365.

[16]	F. Zhou, L. Liu, D. Dai, Z. Huang, Y. Han, J. Huang, Y. Yang, Y. Zou, S. Guo, X. Zhao, P. Li, X. Li, J. Nan, *J. Colloid. Interface Sci.* **2025**, *680*, 364.

[17]	M. Li, W. Li, Y. Hu, A. A. Yakovenko, Y. Ren, J. Luo, W. M. Holden, M. Shakouri, Q. Xiao, X. Gao, F. Zhao, J. Liang, R. Feng, R. Li, G. T. Seidler, F. Brandys, R. Divigalpitiya, T. K. Sham, X. Sun, *Adv. Mater.* **2021**, *33*, e2101259.

[18]	Y. Li, B. Zhang, M. Cao, X. Liang, K. B. Tan, S. Zhang, Y. Dong, Y. Wang, Y. Zhang, H. Gong, H. Rong, A. Dong, X. Han, F. Jin, J. Sun, *Mater. Horiz.* **2025**, *12*, 3420.

[19]	L. Sun, Y. Zhang, D. Zhang, J. Liu, Y. Zhang, *Nano Res.* **2018**, *11*, 2733.

[20]	J. Zhou, X. Liu, W. Cai, Y. Zhu, J. Liang, K. Zhang, Y. Lan, Z. Jiang, G. Wang, Y. Qian, *Adv. Mater.* **2017**, *29*, 1700214.

[21]	W. Kong, W. Li, W. Yu, H. Zhu, S. Xu, S. Liu, L. Cui, Z. Wen, *J. Mater. Chem. A* **2024**, *12*, 4835.

[22]	Y. Zhang, S. Zhang, Y. Cao, H. Wang, J. Sun, C. Liu, X. Han, S. Liu, Z. Yang, J. Sun, *Nano Lett.* **2022**, *22*, 1795.

[23]	W. Kong, Z. Wen, Z. Zhou, G. Wang, J. Yin, L. Cui, W. Sun, *J. Mater. Chem. A* **2019**, *7*, 27048.

[24]	C. Yan, H. Zhao, J. Li, H. Jin, L. Liu, W. Wu, J. Wang, Y. Lei, S. Wang, *Small* **2020**, *16*, e1907141.

[25]	Y. Zhang, L. Wang, H. Xu, J. Cao, D. Chen, W. Han, *Adv. Funct. Mater.* **2020**, *30,* 1909372.
